# Supplementary figures and images for: No Consistent Evidence for Microbiota in Murine Placental and Fetal Tissues
Source: mSphere. 2020 Feb 26;5(1):e00933-19. doi: 10.1128/mSphere.00933-19 (PMC7045391; doi:10.1128/mSphere.00933-19)

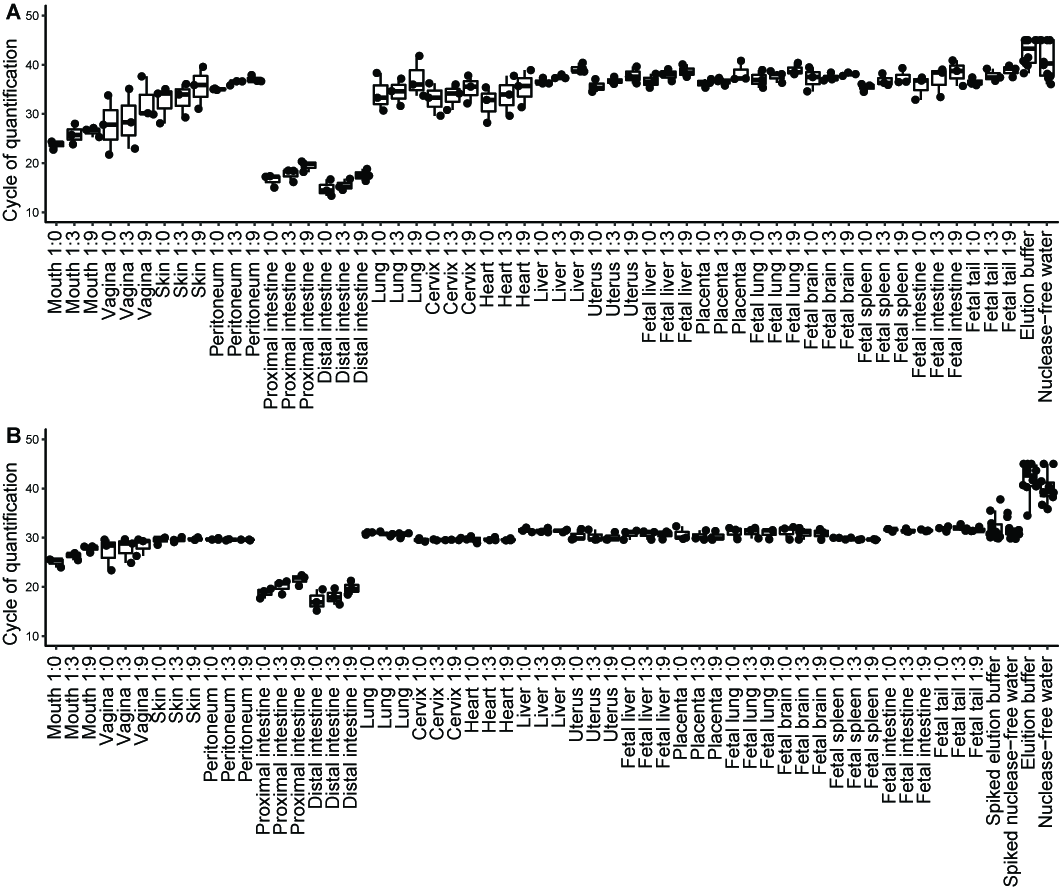

Supplement: FIG S1 [file mSphere.00933-19-sf001.tif]
